# RNA Therapeutics: Delivery Problems and Solutions—A Review

**DOI:** 10.3390/pharmaceutics17101305

**Published:** 2025-10-07

**Authors:** Natalia Pozdniakova, Evgenii Generalov, Alexei Shevelev, Olga Tarasova

**Affiliations:** 1 Blokhin National Medical Research Center of Oncology, Ministry of Health of the Russian Federation, 115522 Moscow, Russia; natpo2002@mail.ru; 2 Faculty of Physics, Lomonosov Moscow State University, 119991 Moscow, Russia; 3 Vavilov Institute of General Genetics of Russian Academy of Sciences, 119991 Moscow, Russia; shevelab@vigg.ru; 4 Institute of Biomedical Chemistry of Russian Academy of Sciences, 119121 Moscow, Russia; olga.a.tarasova@gmail.com

**Keywords:** RNA, RNA vaccine, RNA mimetic, viral vectors, macrophage reprogramming, lipid nanoparticles, AAV vectors, exosomes

## Abstract

RNA-based therapeutics offer transformative potential for treating devastating diseases. However, current RNA delivery technologies face significant hurdles, including inefficient tissue targeting, insufficient selectivity, and severe side effects, leading to the termination of many clinical trials. This review critically assesses the landscape of RNA-derived medicines, examining world-renowned mRNA vaccines (Spikevax, BNT162b2/Comirnaty) and RNA-based therapeutics like Miravirsen (anti-miR-122). It details the composition and clinical trial results of numerous modified short RNA drugs (e.g., siRNAs, miRNA mimetics/inhibitors) targeting various conditions. Prospects for RNA-based medicines are analysed for diseases with substantial societal impact, such as cancer, autoimmune disorders, and infectious diseases, with a focus on evolving delivery methods, including lipid nanoparticles, viral vectors, and exosomes. RNA-mediated macrophage reprogramming emerges as a promising strategy, potentially enhancing both delivery and clinical efficacy. This review highlights that while approved RNA therapies primarily target rare diseases due to delivery limitations, novel approaches in RNA modification, targeted delivery systems, and enhanced understanding of molecular mechanisms are crucial for expanding their application to prevalent diseases and unlocking their full therapeutic potential.

## 1. Introduction

The field of therapeutic development is experiencing a major shift, fueled by the rise in RNA-based therapies that hold promise for treating previously untreatable diseases. While small molecule drugs remain a cornerstone of modern medicine and are widely used, they face inherent limitations. These limitations, including a lack of specificity, difficulty targeting specific molecular pathways, and the high cost and time required for development, leave many diseases–especially hereditary, oncological, and complex viral infections–inadequately addressed by conventional drug regimens. Moreover, these drugs can have significant side effects and toxicity [1,2] and often necessitate long-term or even lifelong treatment [3]. As a result, conditions such as hereditary and oncological diseases, along with certain viral infections, continue to present significant therapeutic challenges for existing drug approaches [4].

Against this backdrop, advances in in vivo RNA delivery and the inherent versatility of RNA molecules have generated excitement about a new generation of RNA-based therapeutics. This innovative approach harnesses RNA’s ability to directly modulate gene expression, offering the potential to create and produce a wide range of new therapies on a single, versatile technological platform [5]. This platform’s capability to streamline drug development and production is seen as a key advantage.

The discovery of RNA interference (RNAi) mechanisms has significantly boosted the development of RNA-based therapeutics [6]. A key advancement was understanding how siRNAs can initiate a potent and sustained therapeutic effect. Unlike mechanisms observed in plants that might involve RNA-dependent RNA polymerase for siRNA amplification [7], the primary amplification mechanism for exogenously delivered siRNAs in mammalian cells relies on the siRNA duplex being unwound within the RNA-induced silencing complex (RISC). The guide strand then directs RISC to the target messenger RNA (mRNA), leading to its cleavage. Critically, the cleaved mRNA is released, and the RISC can then acquire and process additional mRNA molecules, effectively amplifying the silencing signal from a single siRNA molecule [8]. This intrinsic amplification capability suggests the potential to develop nanotherapeutic platforms utilising siRNAs that deliver potent, long-lasting effects at low doses following a single administration.

Another important advantage of RNA-based drugs is their unique ability to directly target different types of signalling pathway regulators: microRNAs (miRNAs) and long non-coding transcripts (lncRNAs) [9].

The first ones who showed that RNAi could be effectively harnessed in mammalian cells were Elbashir et al. [10]. They showed that siRNAs of precisely 21 nucleotides were extremely efficient at acting as mediators of RNAi in cultured mammalian cells. Unlike the specific gene silencing in mammalian cells seen in this work, earlier studies with longer RNA duplexes (>30 nucleotides/strand) were plagued by toxicity and non-specific gene silencing. These unwanted side effects were the result of cytokine induction and protein kinase R (PKR) and toll-like receptor activation. The evidence presented by the researchers that the delivery of these artificial siRNAs of 21 nucleotides could specifically repress target gene expression was crucial as a demonstration of principle for the future use of RNAi in mammals.

The process of developing RNA-based drugs has addressed many issues. In particular, rational siRNA design methods were developed to achieve the drugs with the required pharmacotherapeutic effects [10]; RNA analogues (the so-called mimetics) with high resistance to degradation by nucleases have been created [11]; efficient technologies for the synthesis and delivery of modified RNA were proposed [12]. Researchers have obtained strong evidence supporting the efficacy of therapeutic interventions targeting mRNAs, miRNAs, and lncRNAs with RNA-based therapies [13].

Researchers developed RNA modification techniques to reduce RNA’s sensitivity to nuclease degradation. Modification was essential because native double-stranded RNA (dsRNA) has a half-life of only a few minutes in the bloodstream [14,15]. To maintain the activity of modified siRNA within the RISC, strategies were explored that modified the siRNA itself. These strategies included replacing the phosphodiester bond of the RNA with a phosphorothioate (PS) bond [16] and substituting the 2′ hydroxyl group of ribose—a key moiety for RNase binding—with -O-Me, -O-Et, or -F. Chernikov et al. [17] showed that these modifications do not prevent siRNAs from functioning as inducers of RNA interference. Furthermore, Seth, Tanowitz, and Bennett found that substituting the 2′ hydroxyl of siRNA ribose with -O-Me or -F reduced the substance’s immunogenicity [18].

**Figure 1:**
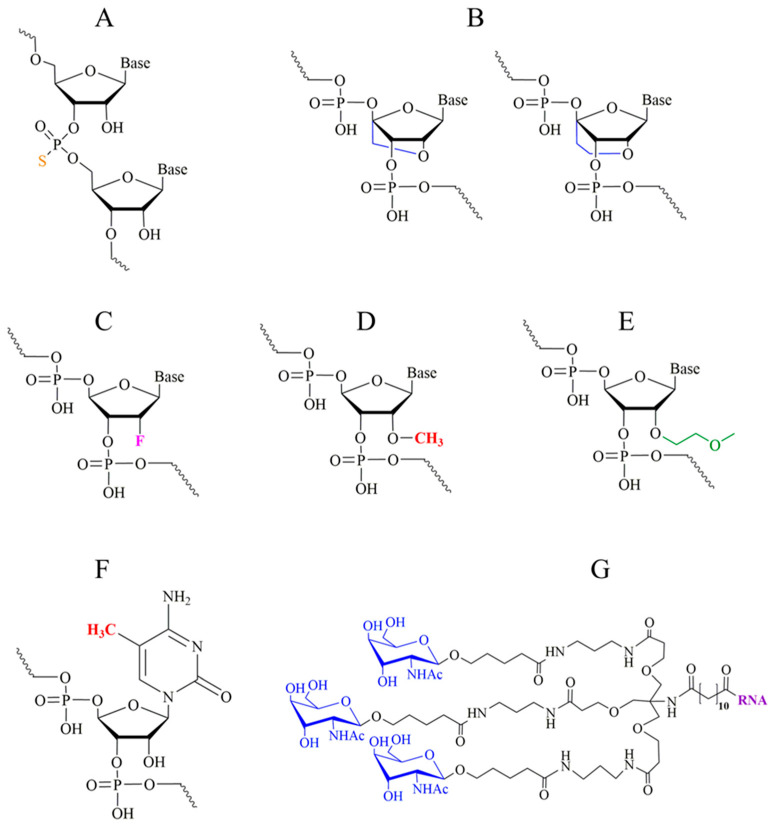
Types of chemical modifications of RNA that occurred in RNA-derived drugs: (**A**) phosphorothioate backbone; (**B**) 2′-O,4′-C-methylene (left) and 2′-O,4′-C-ethylene (right)-ribose (locked sugar, LNA); (**C**) 2′-fluoro-ribose; (**D**) 2′-OMe-ribose; (**E**) 2′-O-(2-methoxyethyl) ribose; (**F**) 5′-methyl-cytosine; (**G**) triantennary GalNAc.

A brief overview of the types of chemical modification of RNA that have occurred in RNA-derived drugs is shown in Figure 1. Among them are the replacement of the phosphate backbone with a phosphorothioate backbone (Figure 1A), modifications of the ribose moiety (Figure 1B–E), and the base (Figure 1F), which provides increased resistance to enzymatic degradation. In addition, the so-called locked nucleic acid (LNA) [19] (Figure 1B), whose ribose residues contain an additional internal bond between the 2′-oxygen and the 4′-carbon, provides improved specificity and base pairing affinity [20]. Finally, RNA can be covalently linked to triantennary N-acetylgalactosamine (GalNAc) (Figure 1G) for targeting purposes.

Some of the modifications could exhibit different negative side effects. For instance, LNA-modified gapmer ASOs can cause significant hepatotoxicity due to their increased affinity, driving off-target RNA degradation by RNase H1. Thus, silencing RNase H1 in the liver or utilising extensive sequence selection and in silico prediction enabled the development of safer and more tolerable LNA-based therapeutics [21].

Currently, the US Food and Drug Administration (FDA), the European Medicines Agency (EMA), and other regulatory authorities have approved several RNA-based drugs [12,22] and two RNA-based vaccines (Comirnaty (Tozinameran) and Spikevax (Elasomeran)) [23]. Most of them are approved drugs that target orphan diseases, which affect a relatively small proportion of the global population. Many other RNA-based drugs have not been approved after years of clinical trials.

This review aims to critically analyse the clinical trial failures of RNA-based therapies, identifying key limitations in target validation and delivery strategies, and proposing a framework based on improved preclinical target validation to enhance the success rate of future clinical trials. This review will explore the advances and persistent challenges of RNA-based medicine, focusing on molecular targets, therapeutic methods, and technological developments in RNA modification and delivery systems. A critical assessment of current successes and roadblocks will be presented.

## 2. siRNA Design Principles and Algorithms

Computational algorithms employed for the selection of siRNA sequences typically integrate a confluence of empirically derived principles. The majority of siRNA design algorithms are designed on the basis of estimating the siRNA binding capacity to the target mRNA and RISC cleavage [24,25]. One of the key parameters for evaluation is thermodynamic stability, which is calculated as the Gibbs free energy of the duplex formation. Most algorithms prioritise sequences with moderate melting temperatures and the absence of complex stable secondary structures in the siRNA molecule. In addition, nucleotides 2–7 of the guide strand in the siRNA primer region are known to correlate with RISC loading and silencing efficiency.

In addition to the intrinsic properties of the siRNA sequence itself, algorithmic considerations are increasingly extended to the characteristics of the target site on the mRNA. The accessibility of a designated target locus within the intricate secondary structure of mRNA exerts a substantial influence upon the binding affinity of siRNA and the subsequent RISC-mediated cleavage. Although accurate mRNA secondary structure prediction is still a computationally challenging task, certain sophisticated design approaches are increasingly integrating procedures to evaluate the availability or accessibility of potential siRNA binding sites. Furthermore, algorithmic considerations are increasingly expanded to include not only the intrinsic properties of the siRNA sequence but also specific features of the target mRNA, such as the presence of conserved nucleotide motifs within the target site or the potential for guide strand binding to be influenced by complementary sequences elsewhere in the mRNA.

In the past, siRNA selection algorithms were mostly based on empirical rules that were extracted from limited experimental data. However, the development of bioinformatics techniques has paved the way for more sophisticated machine learning models. Modern strategies can use algorithmic frameworks, like support vector machines (SVMs), random forests, deep learning models, and convolutional neural networks (CNNs) [26,27].

By being trained on substantial collections of experimentally validated siRNAs, these models are capable of discerning intricate patterns and interdependencies between sequence characteristics and gene silencing outcomes, thereby yielding significantly enhanced predictive accuracy in comparison to antecedent systems. One of the major problems in designing siRNA sequences is the prediction and elimination of off-target effects that can arise as a result of unintended hybridisation of siRNA with off-target mRNAs having a limited degree of homology. To address this exigency, contemporary algorithms integrate sophisticated sequence homology searches across the entire transcriptome or genome of the target organism. Utilities, such as the Basic Local Alignment Search Tool (BLAST) algorithm, are available to identify potential off-target binding sites through the comparison of the designed siRNA sequence against a database of extant sequences. The algorithms then use scoring systems to quantify the likelihood of such off-target binding, taking into account the number and position of mismatches, mismatches in critical regions, and the thermodynamic stability of potential off-target duplexes. There are many predictive algorithms hosted on online siRNA design platforms. For example, BLOCK-iT™ RNAi Designer from Thermo Fisher Scientific is an online tool that has its own algorithms for selecting siRNAs based on predicted efficiency and specificity. Another one is a design tool with predictive algorithms, such as Integrated DNA Technologies (IDTs), to pick up good-quality sequences of siRNA. Also, there are some online tools and software packages that implement these algorithms, such as siRNA Wizard (InvivoGen), siRNA Target Finder (Sigma-Aldrich), siRNA Sequence Finder (Ambion), and siRNA Designer (Thermo Fisher).

Furthermore, researchers frequently employ independent bioinformatics pipelines and bespoke scripts. Such tailored solutions possess the inherent adaptability to integrate novel predictive features or to facilitate the comprehensive screening of prospective targets. For example, specific advanced methodologies leverage machine learning models, which have been rigorously trained on extensive datasets of experimentally validated siRNAs, to prognosticate silencing activity with a high degree of precision [28,29,30].

The fundamental criteria of successful siRNA design are based on the aim to maximise target gene silencing and minimise unspecific binding. The inherent capacity of the siRNA guide strand to be efficiently incorporated into the RISC and subsequently engage with the target mRNA sequence with pronounced specificity and elevated affinity constitutes an indispensable prerequisite for the maximisation of target activity. Therefore, sequences that form a thermodynamically stable duplex with the target sequence are the top priority. Often, the 5′ end of the siRNA, which is critical for initial binding, is the highest priority. Sequences with strong internal secondary structures or low melting temperatures with the target mRNA are usually not used.

At the same time, modification patterns play an important role in regulating biological activity and thus success in siRNA design. The modifications are primarily for increasing the stability, delivery, and specificity and for reducing the immunogenicity of siRNAs.

One crucial modification is 2′-O-methylation (2′-OMe), and it has a great effect on the 3′ terminus of siRNA oligonucleotides. The modification, where the 2′-hydroxyl group that naturally exists on the ribose sugar moieties gets methylated, enables endogenous siRNAs to be identified as “self” and thereby inhibits the induction of innate immune responses that would result if exogenous double-stranded RNAs were present. For chemically synthesised siRNAs, the addition of 2′-OMe greatly decreases immunogenicity by hindering the activation of TLRs and RIG-I-like receptors and thereby diminishing undesirable inflammation reactions. 2′-OMe increases stability as well by shielding the siRNA against being cleaved by 3′-exonucleases and thereby improving its half-life within the biological context and facilitating extended-duration gene silencing.

One very common and effective modification is the addition of a 2′-Fluoro (2′-F) group. The exchange of the 2′-hydroxyl group for a fluorine moiety provides notable nuclease protection and thus increases siRNA stability. The modification may also lead to reduced off-target activity and subtly alter the binding ability of the siRNA, influencing its interaction with its target mRNA as well as the Argonaute 2 (AGO2) protein at the RISC [17,18].

To improve the pharmacokinetic properties and stability of siRNAs, researchers often incorporate PS linkages [16]. The procedure involves the replacement of a non-bridging oxygen atom in the phosphate skeleton with a sulphur molecule. The incorporation of PS linkages enhances the resistance to nuclease and proteolytic degradation for siRNA and, consequently, increases its duration of circulation and its biodistribution. Though beneficial for stability, PS linkage sometimes leads to increased non-specific binding or the triggering of a complement cascade and thus must be optimised carefully.

To obtain greater potency and target less accessible mRNA sequences more efficiently, one incorporates modifications in the form of LNA or constrained ethyl (cEt) modifications [19,20]. They constitute a class of bicyclic nucleic acids in which the ribose ring is conformationally restricted. This conformational restriction significantly enhances the binding efficiency of the siRNA to its target mRNA, resulting in greater gene silencing activity. Again, these modifications enhance stability and enable more specific target engagement and hence have the ability to reduce off-targets.

## 3. RNA Therapeutic Target Types and Approaches to Their Delivery

### 3.1. Molecular Targets of RNA Therapeutics

#### 3.1.1. Historical Context and Early Approaches

Initially, the focus of RNA-based therapies involved targeting gene transcripts with dominant mutations responsible for the development of inherited diseases [31], as well as viral RNA [32]. Targeted destruction of such transcripts resulted in therapeutic effects [33]. Before the discovery of RNA interference, delivery of antisense transcripts capable of forming strong complexes with target RNAs was considered a promising method of targeted transcript repression [34]. The delivery of extended RNAs proved to be technically challenging for most models. Furthermore, the appearance of extended double-stranded RNAs in human cells was shown to induce an interferon response, which can have severe consequences for the patient [35].

Modern examples of RNA types and targets in RNA-derived drugs are listed in Table 1.

#### 3.1.2. RNA Interference (RNAi) and siRNA-Based Therapeutics

With the discovery of RNA interference in *Caenorhabditis elegans* [62] and a similar phenomenon in humans and other mammals [63], it became possible to use siRNAs (short double-stranded RNAs with a chain length of 21 nt each) to suppress gene expression (silencing) [64]. The structural requirements for the siRNA’s structures were developed. It was demonstrated that the preparation of such molecules in accordance with these requirements ensured their incorporation into the Ago2/RISC as guides for the recognition and degradation of target RNAs [65]. Nevertheless, it was determined that a significant proportion of siRNAs developed in accordance with these requirements exhibited only a weak downregulation of a target gene expression in practice [66]. This effect was associated with the need to replenish the intracellular siRNA pool with Dicer nuclease [67]. Subsequently, heuristic criteria for optimising siRNA structure considering Dicer cleavage specificity were derived, and methods for the preparation and screening of siRNA libraries were developed to allow experimental selection of the most efficient siRNAs [68]. The development of this field of RNA-based therapeutic discovery has resulted in the selection of a number of siRNAs against important targets, which have been further transformed into the form of mimetics with increased resistance to nucleases. This approach was implemented by Alnylam Pharmaceuticals in the development of Patisiran, Vutrisiran, Givosiran, Lumasiran, Nedosiran, Inclisiran, Fitusiran and Cemdisiran, as well as by Quark Pharmaceuticals in the development of Teprasiran and Cosdosiran, and by Sylentis S.A. in the development of Tivanisiran [12].

Additionally, in some cases, siRNA libraries are constructed in vitro by cleavage of extended RNA duplexes by purified Dicer nuclease. These RNAs are then injected into cells to address experimental challenges related to the downregulation of target gene transcription [69]. In experiments such as the TNF library, different clones showed varying degrees of DsRed protein reduction when individual DsRed-specific siRNAs were co-expressed with the DsRed vector. This approach maximises the efficiency of RNA interference in a specific object through the autoselection of the most effective siRNAs. Preparatively purified Ago2 nuclease can be introduced into the in vitro system [70]. In particular, Ago2 ensures the selection of the most efficiently incorporated variants of guide RNA as well as the protection of these RNAs from degradation during their delivery to target cells in vivo.

#### 3.1.3. MicroRNA (miRNA) Therapeutics: Mimics and Inhibitors

The difficulties encountered in selecting the most effective siRNA for a given transcript led the scientific community and RNA-based drug developers to consider miRNAs. These are small RNAs of natural origin that are involved in gene silencing using the mechanism of RNA interference. The use of natural guide RNAs to activate the Ago2/RISC ensures its high efficiency. A number of pharmaceutical companies created miRNA mimetics (miR) with enhanced nuclease resistance: Remlarsen/MRG201 (miRagen), TargomiR (EnGeneIC Limited) and MRX34 (Mirna Therapeutics).

In addition, studies on the expression profiles of natural miRNAs and their targets have shown that these molecules themselves may possess significant therapeutic potential due to suppression or activation of expression. Thus, for most types of breast cancer, miR-873 has been shown to be a potent oncosuppressor by downregulating KRAS (Kirsten rat sarcoma virus) oncogene expression [71]. In contrast, miR-484 is an oncogene that suppresses the expression of the oncosuppressor Homeobox protein (HoxA5) [72]. MiR-329-3p [73] and miR-603 [74], miR-22-3p [75] and miR-34a [76] are able to inhibit metastatic growth and can induce the apoptosis of breast cancer cells. MiR-155-5p [77], miR-221-3p [78] and miR-487a [79] have an opposite effect, i.e., they are oncogenic. MiR-199b-5p [80] suppresses neoangiogenesis in the tumours and metastases of breast cancer, and miR-27a [81] and miR-141 [82] induce the growth of new blood vessels in tumour tissue. The development of technology for the synthesis of the LNA (Figure 1), which forms abnormally strong complexes with RNA that cannot be disrupted under physiological conditions, facilitated the design of miR action inhibitors, namely miR-capturer drugs. Such types of drugs include Miravirsen (Santaris Pharma), Cobomarsen (miRagen Therapeutics), and miR-221 inhibitor [83], Lademirsen/RG012 (Regulus Therapeutics), and MRG110 (miRagen Therapeutics).

Despite promising results from in vitro and preclinical animal studies, miR-targeted drugs have exhibited limited efficacy at low doses and an unacceptable risk of adverse effects and toxicity at higher doses during clinical trials. Consequently, none have been approved for clinical use. It is hypothesised that these outcomes stem from insufficient targeting of delivery systems to target cells and an incomplete understanding of the molecular mechanisms of the investigated miRs. It can lead to unintended downregulation of essential genes in non-target tissues and organs, resulting in unpredictable biological consequences.

#### 3.1.4. mRNA Vaccines and Therapeutic Applications

In addition to downregulating the expression of abnormally functioning genes, the delivery of RNA-based pharmaceuticals can be used for gene expression of vaccine antigens. This approach was realised in the development of the Spikevax (Moderna) and BNT162b2/Comirnaty (Pfizer/BioNTech/Fosun Pharma) vaccines [84,85,86,87]. These vaccines are designed to deliver mRNA encoding the SARS-CoV-2 spike protein (S-protein), triggering an immune response against the virus. This approach allowed new vaccines to be created and practically used within two to three months, demonstrating a significant advantage over traditional vaccine platforms. Additionally, mRNA vaccines have certain advantages, such as the potential for rapid development and production, lower risk of insertional mutagenesis than viral vector vaccines, and stimulation of humoral and cellular immune responses. Application of such an approach also led to a decrease in the immunogenicity of the molecule, reduced the possibility of its recognition by TLRs, and increased the efficiency of translation. Both vaccines were produced and used in hundreds of millions of doses. This example demonstrates the advantages of RNA vaccines, which have allowed new vaccines to be created and practically used within two to three months. Such short timeframes are not possible using other known platforms for creating new vaccines.

Despite statistically proven safety [88], it should be noted, however, that in a number of cases, serious adverse effects were observed when using Spikevax and BNT162b2/Comirnaty vaccines [89]. This includes a high risk of myocarditis, a dramatic rise in SARS-CoV-2 infection [90], autoimmune hepatitis [91,92], and neurological complications, and also a dramatic rise in SARS-CoV-2 infection [93]. The reasons for the safety issues are not fully understood, but some considerations exist. For instance, Ioannis P. Trougakos et al. [89] proposed that vaccination-mediated adverse effects may be linked with natural characteristics of the main spike (S) protein of the SARS-CoV-2 virion. S-protein potentially can have properties of molecular mimicry with other human proteins and can bind ACE2 receptors as a ligand.

Cationic lipids from lipid nanoparticles (LNPs) may facilitate adverse effects due to the direct cytotoxic effect [94]. Also, the innate immunity system may be involved in response to the introduction of foreign transcripts [95].

#### 3.1.5. Other RNA-Based Approaches

Regarding the delivery of RNA-based drugs, attention must be paid to the organs, tissues and cell types to which the active ingredient is delivered [96]. Existing systems allow systemic delivery of RNA only to the liver through the portal vein system and receptors that are able to capture RNA. It is also possible to deliver RNA to the kidneys due to hydrodynamic effects arising during primary filtration and to the vascular endothelium due to the longest possible contact with molecules circulating in the vessels [97]. It is important to note that RNA can be delivered to the tumour tissue as a result of the enhanced permeability and retention effect (EPR), which, in turn, is related to the fenestration of blood vessels developed as a result of neoangiogenesis [98]. Topical application can deliver RNA to mucous membranes, primarily the surface of the upper respiratory tract [99], and into the vitreous body of an eye [100]. Macrophages, including tumour-associated macrophages (TAMs), may be another promising target for RNA delivery. The peculiarity of macrophages is the increased phagocytic activity in comparison with other cell types, which can be efficiently used for RNA targeting. This ability can be enhanced by covalently linking RNA to ligands for which receptors are present on macrophages (mannose dimers) [101] or by using a corpuscular form optimal for phagocytosis (use of medium-sized nanoparticles) [102]. The efficacy of RNA targeting may be increased using natural biopolymers that have a high affinity for macrophages (e.g., calreticulin protein) [103,104].

The most important biological feature of TAMs is that, although they make a crucial contribution to the protection of tumour cells from destruction by multiple immune system mechanisms, if their behavioural pattern is changed so that they are able to destroy these cells, they are also able to induce rapid tumour elimination with minimal adverse effects [105]. According to the review [106], there are currently more than 200 TAM reprogramming agents described in the literature with more than 700 clinical trials. Beyond targeting STAT3 and STAT6, other TAM reprogramming strategies include stimulating M1 polarisation using agents like IFN-gamma, blocking immunosuppressive signals such as PD-L1, or enhancing phagocytosis of tumour cells.

The alliance of AstraZeneca (the United Kingdom, UK) and Ionis Pharmaceuticals (the United States of America, USA) developed the original drug Danvatirsen, which is an antisense closed-loop oligonucleotide complementary to STAT3 mRNA [36]. This oligonucleotide is a member of the LNA family and has PS bonds in the backbone. Even with only one or two phosphorothioate bonds per molecule of Danvatirsen, it is highly resistant to plasma nucleases and, importantly, does not require a specific targeting ligand to penetrate the membrane of TAMs. The developers believe that it is a key in reducing off-target RNA interference effects. Danvatirsen suppresses STAT3 not through RNA interference but rather through the stable formation of a heteroduplex between the LNA oligonucleotide and its target mRNA, demonstrating a higher binding affinity compared to conventional RNA duplexes. In the study by T.A. Proia [107], it was shown that Danvatirsen is able to stimulate proinflammatory responses of macrophages and thereby inhibit cancer cell proliferation. Efficacy was observed as a therapy for diffuse large B-cell lymphoma [108]. A Phase 2 clinical trial, completed by July 30 in 2024, evaluated Danvatirsen’s efficacy in a range of malignancies, including bladder cancer, rectal cancer, head and neck tumours, malignant ascites, non-small cell lung cancer, pancreatic cancer, acute myeloid leukaemia, non-Hodgkin’s lymphoma, myelodysplastic syndrome, and various solid tumours. In this trial, no objective responses were noted [37].

**Figure 2:**
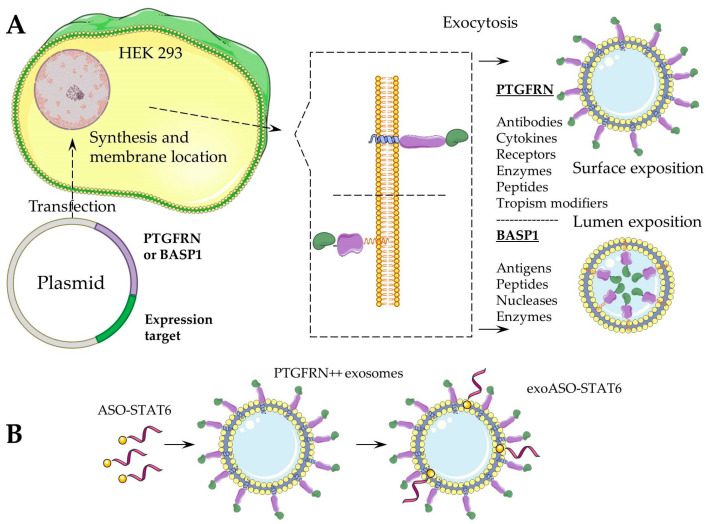
The technology for obtaining engEx exosomes (**A**) and exoASO-STAT6 (**B**).

Codiak BioSciences (the United States) [38] described exoASO-STAT6 (exosomes), which is an LNA-type antisense oligonucleotide designed to capture and exclude the STAT6 mRNA factor from transcription in TAMs. In syngenic models of colorectal cancer and hepatocellular carcinoma, exoASO-STAT6 monotherapy led to 90% inhibition of tumour growth and remission in 50–80% of cases. Administration of exoASO-STAT6 led to induction of nitric oxide synthase 2 (NOS2) production in TAMs. NOS2 is a marker of M1 macrophages, resulting in remodelling of the tumour microenvironment and development of an adaptive immune response mediated by CD8+ T cells. The peculiarity of this approach to targeted suppression of expression is that it does not rely on the canonical endogenous RNAi mechanisms (e.g., the Dicer–RISC pathway), and translation of the corresponding RNA is suppressed due to the formation of a particularly strong complex with a LNA-containing oligonucleotide, leading to steric hindrance. exoASO-STAT6 uses the engEx platform of engineered exosomes as a carrier [109]. engEx exosomes are derived from HEK293 cells transfected with genetic constructs expressing one of two scaffold proteins fused to a therapeutic protein. Previously, K. Dooley et al. [110] identified these two “scaffold” proteins, prostaglandin receptor negative regulator protein F2 (PTGFRN) and brain acid soluble protein 1 (BASP1), which are preferentially sorted into extracellular vesicles (EVs) and provide high-density surface display (PTGFRN) or luminal (BASP1) loading of a wide range of molecules, including cytokines, antibody fragments, RNA-binding proteins, vaccine antigens, Cas9, and members of the TNF superfamily (Figure 2A).

To obtain exoASO-STAT6, PTGFRN++ exosomes were utilised, into which cholesterol-conjugated ASO-STAT6 was incorporated through a mixing process (see Figure 2B).

In addition to TAM, another target of the tumour environment is immunosuppressive myeloid cells. In the study by D. Sarker and co-authors [39], the results of phase 1 clinical trials of the MTL-CEBPA drug are reported. MTL-CEBPA is a double-stranded oligonucleotide of the small activating RNA (saRNA) class that activates the expression of transcription factor C/EBP α (CCAAT/enhancer-binding protein). J. Voutila and colleagues [111] outlined the principle of gene expression activation by Ago2, alongside a co-translational guide RNA that structurally resembles siRNA. NOV340 nanoparticles (SMARTICLE) were used to deliver CEBPA-51 double-stranded oligonucleotide as part of a sense and antisense chain of 21 nt each containing 2′-OMe-modified ribose residues and one PS bond connecting 3′-protruding 2dT residues. Clinical trials enrolled adult patients with advanced hepatocellular carcinoma (HCC) or liver metastases, where the presence of cirrhosis or non-alcoholic steatohepatitis (NASH) was a characteristic of their liver disease. Serious side effects were observed in nine (24%) patients. Of the 24 patients with HCC, sufficient response to tumour treatment was achieved in one patient, and stabilisation was achieved in 12 patients. Therefore, further clinical trials are required to confirm the efficacy and safety of MTL-CEBPA. Subsequently, combination preclinical trials were conducted with encouraging results, with MTL-CEBPA and immune checkpoint inhibitors.

MTL-CEBPA effectively mitigates the immunosuppressive functions of tumour-associated myeloid cells. This action consequently potentiates the efficacy of several therapeutic interventions, namely T-cell targeting therapies, cancer cell inhibitory therapies, PMN-MDSC inhibitory therapies, and radiofrequency ablation. Moreover, triple combination studies have amplified synergistic effects. Thus, the administration of MTL-CEBPA with either an anti-PD-1 antibody and radiofrequency ablation or with an anti-CTLA4 antibody and a COX-2 inhibitor showed significant synergistic effects [112].

### 3.2. Non-Viral Systems for RNA Delivery

**Figure 3:**
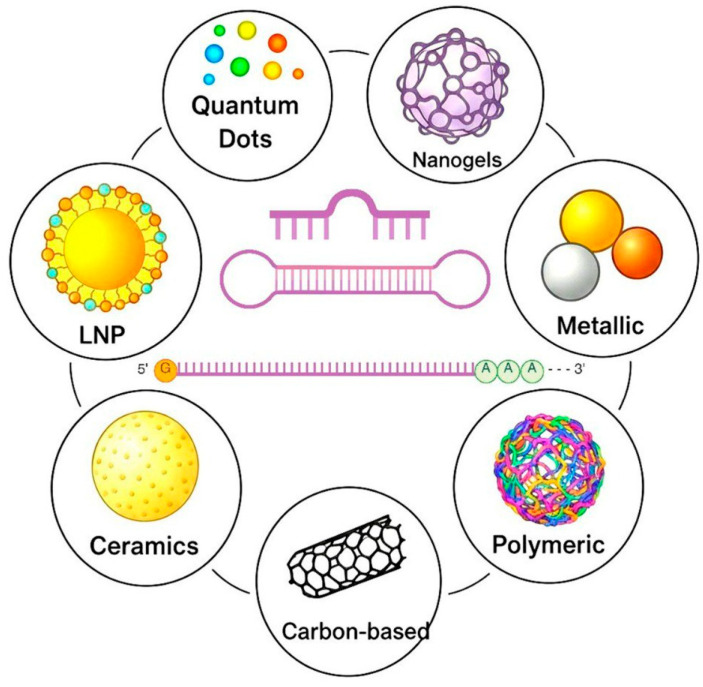
Types of nanoparticles summary.

The use of nanoparticles (Figure 3) that completely prevent interaction with hepatocyte receptors remains another way to increase the duration of RNA circulating in the blood. Depending on their physicochemical properties and surface modifications, nanoparticles can interact differently with the reticuloendothelial system (RES) or mononuclear phagocytic system (MPS) [113,114]. However, it must be taken into account that nanoparticles, especially those with sizes ranging from 10 nm up to a few hundred nanometres and which carry specific surface features such as hydrophobicity or the presence of opsonins, become recognised as foreign material [115,116,117].

Moreover, nanoparticles accumulate within the tumour interstitium, penetrating through the leaky endothelium of tumour vasculature due to hydrodynamic pressure, a process mediated by the enhanced permeability and retention (EPR) effect [118]. TAMs, which express vitronectin and other receptors, also significantly contribute to nanoparticle accumulation within the tumour microenvironment [119]. The EPR primarily facilitates the initial extravasation of nanoparticles from the bloodstream into the tumour. Consequently, the use of larger particles (≥400 nm) can promote prolonged retention within the tumour, albeit with a relatively slow penetration rate [120]. In contrast, small nanoparticles (≤15 nm) may rapidly accumulate in tumours but are often subject to rapid clearance back into the circulation [121]. To enhance the intratumoral retention of small particles, their surfaces can be functionalised with targeting ligands that specifically bind to receptors on tumour cells or TAMs, such as αVβ3-integrins, nucleolin, folic acid receptors, lipoic acid receptors, oestrogen receptors, and luteinising hormone-releasing factor (LHRF) [12]. Medium-sized (especially 100–200 nm) nanoparticles are commonly employed in clinical applications.

The biodistribution of RNA-loaded nanoparticles is strongly influenced by competitive retention within the kidney, liver, vascular endothelium, and tumour tissues, with binding kinetics dictating the relative uptake in each [122]. Consequently, these tissues demonstrate significantly higher nanoparticle accumulation compared to the spleen, lungs, brain, skeletal muscle, glandular tissues, bone, cartilage, ligaments, and mucous membranes, where particle capture is negligible.

The EPR is commonly invoked to explain nanoparticle distribution patterns. However, recent findings highlight the limitations of this paradigm, demonstrating a more pronounced role for the EPR in rodent tumours, including mouse models, than in human malignancies [123]. This may be explained by the higher efficacy and selectivity of nanoparticle-based antitumour agents in trials on human tumour allograft models in immunodeficient mice compared to results obtained in humans during clinical trials [124].

LNPs formulated with lipophilic compounds are the predominant type of nanoparticle employed for RNA delivery, as reported in the literature [125]. In contrast, conventional nanoparticles composed of metals, metal oxides, silicon, carbon, graphene oxide, fullerenes, and quantum dots lack an internal compartment necessary to encapsulate RNA, which is essential for protecting it from nuclease degradation and Kupffer cell clearance [126]. LNPs, in turn, are divided into liposomes, micelles, solid lipid nanoparticles (SLNs), and stable nucleic acid lipid particles (SNALPs). Liposomes for RNA delivery are typically composed of cationic lipids, cholesterol, and polyethylene glycol (PEG) derivatives [127]. To improve the fusion efficiency of liposomes with target cells, the proportion of unsaturated cationic lipids was increased [128]. The incorporation of neutral lipids and phospholipids helps to increase the duration of particle circulation in the bloodstream [129].

The problem of using LNPs for RNA delivery is the insufficient release of the delivered molecule from the complex with transport lipids in liposomes, which may lead to its degradation. To increase the efficiency of RNA release into the cytoplasm from lysosomes, it was proposed to use acid-labile peptide bonds that spontaneously break down in lysosomes under low pH [130]. Use of amphoteric lipids that are anionic or neutral at the pH of the extracellular environment (around 7.0) but can be converted to cationic form at the acidic pH, which is typical for lysosomes [131].

In addition to LNPs, nanoparticles are based on hydrophilic polymers. For instance, polyethyleneimine (PEI) was used for RNA delivery [132]. Also, the usage of chitosan [133], polylactide [134], polylysine [135], and dendrimers [136] is described. Calcium carbonate-based nanoparticles are also applied [137].

Among the materials used for non-viral gene delivery in vitro and in vivo, poly(β-amino ester)s (PBAEs), a class of synthetic biodegradable polymers, have become a leading gene delivery vehicle that has been used for multiple applications [138,139].

PBAEs offer an advantage in nucleic acid delivery due to their inherent positive charge, which facilitates binding to negatively charged nucleic acids. This charge, however, is pH-dependent and can be neutralised at elevated pH levels, as is typical in the extracellular environment. Following cellular uptake, PBAEs undergo rapid degradation, releasing the encapsulated nucleic acids. Nevertheless, this issue can be mitigated through chemical modifications or by incorporating PBAEs into composite materials [140].

Common ligands used for targeted delivery of RNA-containing particles to tumours [141] include mannose dimers (targeting TAMs), RGD (arginylglycylaspartic acid) peptides (ligands for αVβ3 integrins characteristic of tumour vessels) [142], luteinising hormone-releasing hormone [143], folic acid [144], and lipoic acid [145]. To improve the delivery of particles to the hepatocytes, N-Acetylgalactosamine (GalNAc) [146,147] is used.

In addition to the use of delivery systems (for instance, nanoparticles or liposomes), some drugs may use a strategy of direct chemical conjugation of RNA to the targeting ligand. In particular, RNA delivery to the liver is used due to the direct conjugation of the RNA strand with GalNAc (Figure 1) [148]. A similar approach has been applied to the specific bicyclic peptide BiPPB–a ligand of platelet-derived growth factor receptor beta (PDGFβR), which is used to target liver fibrosis [149].

Because of its unique known anatomy and cellular constitution, the liver has become the dominant target for RNAi gene-silencing therapeutics (and one of the key enabling factors is that delivery strategies have progressed so much). The advent of N-acetylgalactosamine (GalNAc)-conjugated therapeutics has changed the landscape, as it has provided an easier way to target specific cells, especially for liver-targeted small interfering RNA (siRNA) technologies. The triantennary GalNAc is chemically linked to the siRNA by attachment via a covalent bond to one strand, usually at the 5′ or 3′ terminal. The nature of the GalNAc-based delivery is based on the high expression of the asialoglycoprotein receptor (ASGPR) on the surface of hepatocytes. ASGPRs are transmembrane receptors that recognise and internalise galactose-terminal glycoproteins. Upon systemic administration, the GalNAc-conjugated siRNA is efficiently captured by hepatocytes through receptor-mediated endocytosis. Upon internalisation into endosomes, the acidic conditions of the endosomal vesicles induce the release of the siRNA, which allows for siRNA to escape into the cytoplasm, join the RNA-induced silencing complex (RISC) and perform its gene-silencing activity. This targeted mechanism significantly enhances siRNA bioavailability in the liver while minimising off-target accumulation in other tissues, thereby improving therapeutic efficacy and reducing systemic toxicity.

An indication of the potency of GalNAc-conjugated siRNAs is the number of approved RNAi therapeutics developed to successfully target genes expressed in the liver. Inclisiran (Leqvio^®^) reduces high low-density lipoprotein (LDL) cholesterol and an increased risk of premature atherosclerotic cardiovascular disease. Vutrisiran (Amvuttra^®^) reduces transthyretin (TTR) protein levels by targeting TTR mRNA to treat hereditary transthyretin-mediated amyloidosis. Givosiran (Givlaari^®^) is an agent that inhibits aminolevulinate synthase 1 (ALAS1) in the liver and is indicated for the treatment of acute hepatic porphyria. Lumasiran (Glycots^®^) is an agent that inhibits oxalate transporter 1 (OXT1) and is indicated for the treatment of primary hyperoxaluria type 1. Nedosiran (Rivfloza^®^) targets the *LDLRAP1* gene and is approved for the treatment of hepatic galactose metabolism and primary hyperoxaluria type 1. Fitusiran, an investigational drug, inhibits antithrombin mRNA for the treatment of haemophilia A and B. Cemdisiran targets C5 complement in the liver and is approved for the treatment of primary hyperoxaluria type 1 and is in development for the treatment of dry age-related macular degeneration. These agents have received clinical approvals that provide evidence for the use of GalNAc-based targeting for therapeutic gain in liver disease [150].

In addition to GalNAc conjugation, liposomal siRNA delivery systems have demonstrated considerable potential for liver-targeting. LNPs contain specific types of ionisable lipids, helper lipids, cholesterol, and PEGylated lipids that encapsulate siRNA, preventing its degradation while allowing for cellular uptake. The formulation of LNPs is important in relation to targeting the liver [151]. There are inherent uptake mechanisms that take place in hepatocytes which, along with interactions with the RES, ApoE-mediated uptake and specific cellular transporters, result in the preferential uptake of LNPs in the liver. While the precise lipid formulations for hepatic uptake will vary, they also generally act via the anionic nature of the cell membrane and will depend on the particle’s overall charge and surface properties. Studies in this area are ongoing and focused on optimising LNP formulations by generating unique ionisable lipids which aim to reduce toxicity and improve endosomal escape, but also aim to optimise typical parameters such as size and surface modification to optimise hepatic accumulation and intracellular delivery [152,153].

The remarkable success achieved in liver-targeting, primarily through GalNAc conjugation and optimised liposomal formulations, starkly contrasts with the persistent challenges in delivering RNA therapeutics to other vital organs and tissues. While LNPs are the primary delivery mechanism for mRNA vaccines, achieving clinically relevant therapeutic concentrations in non-hepatic tissues, such as the brain, lungs, or muscle, will remain a challenge. Viral vectors can efficiently transduce additional cell types but are limited by immunogenicity and tropism [154]. Besides that, for non-viral vectors, specifically any that cross the blood–brain barrier or have the ability to target solid tumours with complete delivery, are ongoing areas of research [155]. By referencing liver-targeting, we demonstrate some validation of the concept; therefore, effective RNA pharmaceuticals exist for diseases beyond the liver. The demand for delivery systems that can complement RNA-based modalities for a wider range of diseases, including non-liver delivery, should be prioritised [156].

Although siRNA and microRNA are both nucleic acids used in therapeutic applications, carriers developed from transporting these nucleic acids generally exhibit considerable differences, with the variations largely based on their different attributes as small and typically double-stranded (siRNA) or processed from hairpin structures (miRNA). The small size of siRNA and miRNA gives an opportunity to pack them into viral vectors or conjugate them to targeting moieties to allow cells to uptake and release the nucleic acids intracellularly to silence gene expression [157,158].

mRNAs are substantially larger single-stranded molecules (1–15 kb) than siRNAs (∼14 kDa), and thus delivering mRNA is more complicated. Because of its larger structure and in order to protect the molecule from degradation, it often uses more sophisticated or larger delivery systems, like LNPs. Moreover, the goal of mRNA delivery requires carrier systems that can not only protect the cargo but also enable it to escape from endosome vesicles and reach cells’ organelles. This is significantly more complex than siRNAs that aim at RNA silencing. The complex architecture of the typical mRNA carriers (ionisable lipids, helper lipids, cholesterol, and PEG-lipids) demonstrates the complexity that must be addressed to deliver a large and functional mRNA payload [159].

Polymeric materials are highly attractive for RNA delivery due to their inherent versatility, stemming from a combination of precisely controllable characteristics and favourable biological compatibility. The profound tunability of polymeric architecture and composition allows for exquisite control over critical physicochemical parameters such as charge density, hydrophilicity–lipophilicity balance, and molecular weight, enabling the optimisation of RNA encapsulation, protection, and intracellular release. Furthermore, the capacity for programmed biodegradability offers a critical solution to concerns of immunogenicity and long-term accumulation, as polymers can be designed to degrade into biocompatible metabolites, facilitating controlled release and minimising toxicity. Finally, the intrinsic potential for sophisticated cell targeting, achieved by functionalising polymer surfaces with specific ligands, allows for active guidance of RNA-loaded nanoparticles to target cells, thereby minimising off-target effects, reducing systemic toxicity, and significantly enhancing the therapeutic index of RNA-based therapeutics, transforming delivery into a precision medicine strategy. Features and types of nanoparticles used in RNA delivery systems are summarised in Table 2.

### 3.3. Viral Vectors for RNA Delivery

Viral vectors produced by recombinant DNA technologies are considered a promising tool for gene therapy due to their high transduction efficiency and ability to ensure prolonged expression of transgenes in various cell types. Lentiviruses (LVs), adenoviruses (Ads) and adeno-associated viruses (AAVs) are the most widely used viral vectors for therapeutic purposes [195,196]. In the LVs group, human immunodeficiency virus (HIV) is the most common, with simian immunodeficiency virus (SIV) and feline immunodeficiency virus (FIV) used to a lesser extent [197,198]. LVs particles have been successfully used to restore miRNA expression [195,199]. Overexpression of miR effectively suppresses proliferation and induces apoptosis in oesophageal cancer cells [200]. However, the ability of retroviruses to insert into the genome of host cells (which can lead to inactivation of genes, genetic instability, the ability to activate oncogenes, and affected histone modification) makes the clinical use of LVs-based vectors extremely risky. Significant efforts have been made to reduce the mutagenic activity of LVs, particularly by removing the sequences responsible for integration into the human genome. One approach involves creating LVs variants that replicate as episomes [201]. However, even in this form, the mutagenic potential of retroviruses remains high. At present, there are practically no perspectives for introducing such vectors into practice for safety reasons [202].

In contrast to LVs-based vectors, third-generation adenovirus-based vectors stably express microRNAs without integrating into the host genome [203]. In the study by X. Bofill-De Ros et al. [204], the authors described the creation of oncolytic viruses using this platform to deliver microRNA-148a to pancreatic cancer cells. The viruses were shown to be completely safe for normal cells in the human body. However, a serious disadvantage of adenovirus-based vectors is their tendency to induce reactions in the innate and adaptive immune systems. To overcome this issue, adenoviruses are modified with polymers. In addition, various ligands are often inserted into their capsids to enhance the tropism of such vectors to tumours [205].

AAVs are the safest and least immunogenic among other types of viral vectors. The AAVs with different variants of tissue tropism make them a promising tool for in vivo gene delivery [206,207]. There are currently 13 known AAV serotypes, most of which have been validated in gene delivery experiments [208]. AAVs are not prone to incorporation into the genome, so they are suitable mainly for transient expression of miRNAs. Bhere et al. [209] showed that co-delivery of miR-21 and miR-7 using AAV-based vectors effectively induced apoptosis in mice with brain tumours. This approach is particularly effective when combined with other cancer therapies. In the study by Bhere D. et al. [210], the authors demonstrated the combined use of an AAV-based vector for miR-7 delivery and S-TRAIL ligand delivery via stem cell (SC) for the treatment of glioblastoma. Exposure of the S-TRAIL ligand to its receptor triggered tumour cell death. Specifically, the authors showed that co-delivery of miR-7 and S-TRAIL using SC potently suppressed tumour growth and increased the survival of mice. Overall, the delivery of AAV-mediated microRNAs holds great promise in cancer therapy. They could be an effective means of clinical application of microRNAs if their induction of immune reactions can be completely overcome.

In the study by Yang Y.S. et al. [211], AAV-based constructs have been reported for the therapy of fibrodysplasia ossificans progressiva (FOP). These constructs contain an insert of the codon-usage-optimised ACVR1 gene and a set of artificially co-engineered siRNAs targeting activin A and its receptor ACVR1R206H. Local administration of the engineered AAV vector to the skeletal muscle of Acvr1R206H/+ mice resulted in decreased endochondral bone formation. The vector showed no expression of the delivered genes in the liver. The authors attribute the AAV’s transgene silencing to repression by miR-122, which is abundant in the liver.

The virus-like particles (VLPs) are non-infectious particles based on proteins from bacteriophages and viruses. Their usage for drug delivery was proposed in the early 1990s [212]. VLPs can be assembled in vitro, then drugs or small molecules can be injected into them [213,214]. Bacteriophage MS2 is a member of the family *Leviviridae*. The host of the bacteriophage MS2 are bacteria of the Enterobacteriaceae family. The MS2 genome is a single-stranded RNA [215,216]. MS2 is typically used to produce VLPs and has many unique properties that make it an interesting candidate for targeted gene delivery, such as rapid acquisition, self-assembly in the presence of nucleic acids, and the ability to insert additional sequences into the capsid protein [217].

In the study by Hoffmann et al. [218], the authors reported the usage of a John Cunningham virus (JC) capsid protein VP1 for VLP construction. JC virus is a member of polyomaviridae that causes central nervous system diseases. This protein is able to autonomously form VLPs both in vitro and in vivo. The advantage of these VLPs based on JC-VP1 over VLPs based on bacteriophages PP7 and MS2 is the ability of such particles to penetrate into neurones of the central nervous system [219].

In general, VLPs are more stable both in storage and in the bloodstream than LNPs and provide good protection of delivered RNA from degradation by nucleases. VLPs are also characterised by low toxicity and safety. VLPs respond to a decrease in pH upon entering lysosomes, which increases their possibility of use as a drug delivery vehicle to target cells. Functional tumour-specific ligands can easily be introduced into VLPs to enhance the targeting of delivery; however, the practicality of this approach depends on the immunogenicity of such ligands in repeated administration [220,221,222].

Types of RNA modifications and delivery systems in RNA-based drugs are summarised in Table 3.

## 4. Discussion

While LNPs have emerged as a mostly used delivery system for RNA therapeutics, significant limitations remain. Manufacturing LNPs with consistently controlled size (typically 50–200 nm) and uniform lipid composition is a complex process. Batch-to-batch variability can impact drug efficacy and toxicity, requiring strict quality control measures. The microfluidic mixing methods employed often demand precise control of flow rates and lipid ratios, raising challenges for large-scale production. Further, the choice of lipid components, particularly the ionisable lipids crucial for endosomal escape, can significantly influence the LNP’s properties and requires careful optimisation.

What is more, a major hurdle is extending LNP delivery beyond the liver, where they naturally accumulate due to ApoE-mediated uptake by hepatocytes. Targeted delivery to other tissues necessitates surface modifications with ligands specific to receptors on target cells. However, achieving high specificity and avoiding off-target uptake remain challenging. PEGylation, often used to prolong circulation time, can also hinder cellular uptake (the ‘PEG dilemma’).

Recently there have been several innovative approaches to overcome these limitations. First of all, the development of novel ionisable lipids with improved efficacy and safety profiles, aiming for better endosomal escape and reduced toxicity. Secondly, targeted ligand display strategies, such as using multivalent ligands or stimuli-responsive linkers to enhance binding affinity and internalisation at target cells. Thirdly, combine LNPs with exosome-derived components to improve biocompatibility and targeting. In addition, LNPs can be designed with a specific shape and stiffness, which improves tissue penetration and cellular uptake. Elongated or deformable particles may be more effective at navigating the vasculature and penetrating tissue.

The potential of viral delivery of regulatory RNA using viral vectors, primarily adeno-associated virus (AAV), remains underutilised. However, this approach faces the challenge of reducing the nonspecific immune response to the virus or reducing the necessary RNA doses. Reducing the required RNA doses could be achieved by amplifying siRNA in vivo with the participation of RNA-dependent RNA polymerase, a process observed during RNA interference in invertebrates [223] and plants [7,224]. Alternatively, improving RNA delivery efficiency by minimising losses during transit through liposomes can be achieved by increasing the use of pH-reactive carriers such as amphoteric lipids and PBAE [138,139].

At the same time, AAVs offer high transduction efficiency, but immunogenicity remains a significant concern. Antibodies against AAV serotypes can neutralise the vector and reduce its therapeutic efficacy. The capsid proteins and nucleic acids of AAVs can also elicit cellular immune responses, leading to inflammation and potential toxicity. Strategies to mitigate these issues include capsid engineering, immunosuppression, co-administration of empty AAV capsids, and the use of microRNA to restrict AAV expression in immune cells.

LVs, while capable of long-term gene expression due to their ability to integrate into the host genome, have the risk of insertional mutagenesis. Although significant efforts have been made to reduce this risk by deleting or modifying viral sequences involved in integration, the potential for LV integration to disrupt tumour suppressor genes or activate oncogenes remains a concern. Also, such insertions raise huge ethical questions. Furthermore, some studies [225,226] have suggested that even self-inactivating LV vectors, which lack the 3′ long terminal repeat (LTR), can still exhibit some level of insertional activity.

Beyond LNPs and viral vectors, cell-derived vesicles, particularly exosomes, are gaining attention as potential delivery systems for RNA therapeutics. However, challenges remain in scaling up exosome production, controlling their cargo loading, and ensuring consistent quality. Genetically engineered cells can be used to produce exosomes with enhanced targeting capabilities and therapeutic payloads. Further research is needed to optimise exosome-based delivery systems and demonstrate their clinical efficacy.

### Future Perspectives

The therapeutic potential of RNA-based modalities in combating viral infections and oncological diseases is exceptionally promising. Existing RNA therapies, exemplified by mRNA vaccines, are anticipated to undergo refinement and expansion. This evolution is projected to extend applications beyond infectious disease prophylaxis to encompass therapeutic vaccines for cancer and the treatment of autoimmune and genetic disorders.

Development of novel RNA delivery systems is paramount. Emerging technologies, including next-generation lipid nanoparticles, engineered extracellular vesicles, and novel polymer-based nanocarriers, are being developed. These are complemented by complex conjugation strategies, such as GalNAc, aptamer, and peptide conjugates. Such approaches aim to overcome challenges associated with side effects, off-target biodistribution, and immunogenicity.

These advancements are poised to facilitate enhanced tissue specificity, thereby reducing systemic toxicity. Breakthroughs in targeted delivery are anticipated via receptor-mediated strategies, microenvironment-responsive systems, and immune cell targeting. Also, this could be used in personalised medicine, enabling patient-specific RNA design, biomarker-driven therapy selection, and adaptive combinatorial treatment regimens.

This convergence of sophisticated delivery platforms, targeted molecular action, and individualised patient data portends a new era of highly effective and safe RNA-based therapies for a broad spectrum of diseases. Nevertheless, navigating complex manufacturing processes and stringent regulatory pathways will remain critical for successful clinical translation.

Furthermore, integrating RNA therapies with established treatment modalities holds considerable promise. Combining RNA-based interventions with immunotherapies, radiotherapy, and conventional chemotherapy may elicit synergistic effects. Concurrently, novel strategies are emerging for developing combinations of different RNA therapies. These can be engineered to exert synergistic effects specifically within target cells.

As experience with RNA therapies accrues and understanding of intricate cellular regulatory networks deepens, developing more precise and personalised RNA-based treatments will become increasingly feasible. Preliminary molecular biological analysis (including sequencing, transcriptome, hyperexpression, etc.) of patients’ biomaterials will allow targeted programming of RNA for the most effective therapy of the target disease (oncology, viral infection, autoimmune diseases, etc.). This trajectory will ultimately pave the way for a new generation of highly effective and safe therapeutics capable of addressing a broad spectrum of pathologies.

## 5. Conclusions

RNA-based therapeutics offer a groundbreaking approach to disease treatment by directly influencing gene expression. The rapid success of mRNA vaccines during the COVID-19 pandemic has highlighted their immense potential, and RNA interference (RNAi) drugs like Patisiran have already demonstrated the feasibility of treating rare diseases. However, realising the full therapeutic impact of these drugs requires overcoming significant hurdles.

A primary bottleneck lies in RNA delivery. While current technologies, particularly LNPs, are advancing quickly, they still face limitations. LNPs, the most common delivery vehicle, struggle with consistent manufacturing quality, extending delivery beyond the liver, and penetrating the blood–brain barrier. Viral vectors, especially AAVs, offer high transduction efficiency but are limited by immunogenicity. Lentiviral vectors, another viral option, carry the risk of insertional mutagenesis. Promising emerging strategies, such as cell-derived vesicles, require further optimisation to achieve scalable production and precisely control their cargo loading.

The future of RNA therapeutics depends on successfully addressing these delivery challenges, alongside continued advancements in RNA chemistry and a more thorough understanding of disease pathways. By integrating these advancements, we can unlock the full potential of RNA-based medicines and broaden their application to a wider spectrum of prevalent diseases, ultimately reshaping modern medicine. The prospect of personalised, targeted, and effective RNA therapies is becoming increasingly tangible, and sustained research and innovation are crucial to realising this transformative vision.

## Figures and Tables

**Table 1:** Examples of RNA types and targets in RNA-derived drugs.

RNA Type/Mechanism of Action	Drug Name	Molecular Target	Therapeutic Application	References
ss ASO (single-strand antisense oligonucleotide) induces gene silencing	Danvatirsen	STAT3	Therapy of bladder, colorectal, pancreatic cancer, non-small cell lung cancer (NSCLC), head and neck tumours, malignant ascites cancer, acute myeloid leukaemia at the stage of non-Hodgkin’s lymphoma, myelodysplastic syndrome	[36,37]
	exoASO-STAT6	STAT6	Therapy of advanced hepatocellular carcinoma (HCC), liver metastases from either primary gastric cancer or colorectal cancer (CRC) and oral squamous cell carcinoma	[38]
saRNA (small activating RNA) target gene promoter regions and enhance the transcription of a desired gene	MTL-CEBPA	C/EBP-α	Combined therapy of hepatocellular carcinoma (HCC), cirrhosis of the liver, non-alcoholic steatohepatitis and liver metastases	[39]
miR inhibitor binds to and inhibits miRNAs involved in the pathogenesis process	Cobomarsen/MRG-106	miR155	Therapy of T-cell lymphoma (CTCL) [mycosis fungoides (MFs) subtype], chronic lymphocytic leukaemia (CLL), diffuse large B-cell lymphoma (DLBCL)	[40]
	Miravirsen/SPC3649	miR-122 (HCV 5′-UTR)	Chronic hepatitis C therapy	[41]
	LNA-i-miR-221	miR-221 (CDKN1B/p27 and PTEN)	Refractory Multiple Myeloma, HCC	[42]
	MRG110	miR-92	Wound healing and preventing heart failure	[43]
	Lademirsen/RG012	miR-21	Treatment of Alport syndrome	[44]
mRNA Antigen synthesis and presentation for immunisation	Spikevax	Spike protein	Prophylaxis of SARS-CoV-2 infection	[45]
	BNT162b2/Comirnaty	Spike protein	Prophylaxis of SARS-CoV-2 infection	[46]
ds miR-mimetic mimics lost oncosuppressive function miRs	TargomiR	miR-16	Treatment of refractory malignant pleural mesothelioma (MPM) and lung cancer (NSCLC)	[47]
ds miR-mimetic	MRX34	miR34 (MET, MYC, PDGFR-α, CDK4/6, BCL2, PD-L1, DGKζ)	Melanoma, lymphoma, renal cell carcinoma, multiple myeloma, NSCLC, primary liver cancer, SCLC	[48]
	Remlarsen/MRG201	miR-29 (collagens)	Prevention of fibrous and keloid scar formation	[49]
siRNA suppresses the expression of key genes in disease pathogenesis through mRNA degradation	Patisiran/Onpattro/ALN-TTR02	Transthyretin (TTR)	Treatment of hereditary transthyretin amyloidosis (familial amyloid polyneuropathy) (hATTR)	[50]
	Vutrisiran/Amvuttra	Transthyretin (TTR)	Treatment of hATTR	[51]
	Givosiran/Givlaari	δ-aminolevulinate synthase 1 (ALAS1)	Treatment of the acute hepatic porphyria (AHP)	[52]
	Lumasiran/Oxlumo	Glyoxylate oxidase (GO)	Therapy of primary hyperoxaluria type 1 (PH1)	[53]
	Nedosiran/Rivfloza	Lactate dehydrogenase	Therapy of all types of primary hyperoxaluria	[54]
	Inclisiran/Leqvio	Proprotein convertase subtilisin/kexin type 9 (PCSK9)	Therapy for high low-density lipoprotein (LDL) cholesterol and an increased risk of premature atherosclerotic cardiovascular disease	[55]
	Fitusiran	Antithrombin	Therapy of haemophilia A or B	[56]
	Teprasiran	p53 antioncogene	Therapy of delayed graft function (DGF)	[57]
	Cosdosiran	Caspase 2	Therapeutic for the nonarteritic anterior ischaemic optic neuropathy (NAION) and the primary angle closure glaucoma	[58]
	Tivanisiran	TRPV1 receptor	Treatment of dry eye disease	[59]
	Cemdisiran	Complement Component C5	Therapy of immunoglobulin A nephropathy (IgAN), paroxysmal nocturnal haemoglobinuria, myasthenia gravis, atypical haemolytic uremic syndrome	[60,61]

**Table 2:** Non-viral nanoparticles used in RNA delivery systems.

Nanoparticles	Design Principal	Mechanism of Action	Advantages	Limitations	Reference
LNP	Chemical (the combination of the ionisable lipid, PEG-lipid, cholesterol, and a helper lipid) or biogenic particles (exosomes) from 50 to 200 nm	RNA electrostatic complexation and encapsulation. Clathrin-mediated endocytosis, receptor-mediated uptake, fusion with endosomal membrane	Efficacy and safety, RNA protection, effective endosomal escape, nontoxic, biocompatible, tunable formulation, longer circulation time, targeting capability, definite structure	Poor interaction with RNA for neutral lipids, toxicity and immunogenicity for cationic lipids, aggregation, high costs	[160,161,162,163,164]
Nanogels	Three-dimensional networks of cross-linked hydrophilic, natural, or synthetic polymers from 50 to 200 nm	Electrostatic interactions, physically trap RNA, covalent conjugation, and hybrids with other RNA carriers. Clathrin- or caveolae-mediated endocytosis, receptor-mediated uptake	High loading capacity, stability, stimuli-responsiveness, biocompatibility and biodegradability, protection of RNA, tunable properties, prolonged release, no need for multiple administrations	Complexity of synthesis, immunogenicity, non-specific uptake, aggregation	[165,166,167,168,169]
Metallic	Metall (Au, Al, Pt, Zn, Fe, etc.) particles from 1 to 100 nm with a high surface-area-to-volume ratio and quantum mechanical effects	Electrostatic interactions, physically trap RNA. Clathrin- or caveolae-mediated endocytosis, macropinocytosis, and targeted uptake, magnetic targeting	Versatility, high electrical and thermal conductivity, unique stimuli-responsive properties, biocompatibility, strong electrostatic interactions with RNA	Low thermal and corrosion resistance, disrupting multiple cellular functions, proinflammatory, activating/inhibiting different pathways, low biodegradability	[170,171,172,173,174]
Polymeric	Natural, synthetic, or semi-synthetic polymers from 1 to 1000 nm	Electrostatic interactions, physically trap RNA. Clathrin- or caveolae-mediated endocytosis, macropinocytosis, targeted uptake. Endosome escape. RNA can be linked via pH-cleavable linkers	Versatility, tunable properties, biocompatibility and biodegradability, low toxicity, efficient RNA complexation, endosomal escape, easy to functionalise	Polymers can be cytotoxic, immunogenic, off-target delivery, and endosomal escape	[175,176,177,178,179,180]
Carbon-based	Carbon particles from 1 to 100 nm (single-walled or multi-walled carbon nanotubes; 2D sheets of graphene or graphene oxide with large surface area; fullerenes; carbon dots; amorphous carbon nanoparticles; graphyne; nanodiamonds; carbyne)	Physically encapsulating through hydrophobic interactions and π–π stacking, cell membrane penetration and endocytosis release	Electrical and thermal conductivity, mechanical strength, high stability, high surface area, adjustable fluorescence	Aggregation, insolubility, non-biodegradability, agglomeration, immunogenicity, high cost, carcinogenicity, oxidative stress	[181,182,183,184]
Ceramics	Inorganic, nonmetallic particles (carbonates, phosphates, oxides, carbides, TiO_2_, Si_3_N_4_, etc.) up to 200 nm. Porous core, functionalised surface with charge-modifying groups or biocompatible polymers	Electrostatic interactions, physically trap RNA. Clathrin- or caveolae-mediated endocytosis, macropinocytosis, targeted uptake	Deeper penetration into capillaries, fenestrations, high heat and corrosion resistance, chemical stability, diverse electrical properties	Brittleness, disrupting multiple cellular functions, proinflammatory, activating/inhibiting different pathways, endosomal escape	[185,186,187]
Quantum dots	Semiconductor nanocrystal complexes from 10 to 100 nm synthesised with a core from 2 to 10 nm (CdSe, CdTe), a shell (ZnS) and a functionalised surface	Electrostatic interactions, RNA covalent conjugation or secondary carrier encapsulation within a. Clathrin- or caveolae-mediated endocytosis, macropinocytosis, targeted uptake, co-delivery	Bright, stable, and tunable fluorescence, cellular entry, dual-modality (carrying and tracing)	Cytotoxic, need of endosomolytic components for endosomal escape, immunogenicity	[188,189,190,191,192,193,194]

**Table 3:** Examples of RNA modification types in RNA-derived drugs.

Delivery System	Phosphorothioate Backbone				Phosphodiester Backbone	
	2′-F and 2′-OMe Ribose	2′-F, 2′-OMe Ribose, and dT	2′-MOE, 2′-F, and 2′-OMe	LNA	2′-F and 2′-OMe Ribose	No Modifications
LNP		Patisiran MTL-CEBPA				
HEK-293-derived exosomes					exoASO-STAT6	
*E. coli*-derived exosomes						TargomiR
Triantennary GalNAc covalent modification		Vutrisiran Givosiran Lumasiran Nedosiran Fitusiran Cemdisiran	Inclisiran			
No	Miravirsen Remlarsen			Danvatirsen LNA-i-miR-221	Teprasiran Cosdosiran	Tivanisiran

## Data Availability

No new data were created or analyzed in this study. Data sharing is not applicable to this article.
